# Using GPS Collars and Sensors to Investigate the Grazing Behavior and Energy Balance of Goats Browsing in a Mediterranean Forest Rangeland

**DOI:** 10.3390/s22030781

**Published:** 2022-01-20

**Authors:** Youssef Chebli, Samira El Otmani, Jean-Luc Hornick, Abdelhafid Keli, Jérôme Bindelle, Mouad Chentouf, Jean-François Cabaraux

**Affiliations:** 1 Regional Center of Agricultural Research of Tangier, National Institute of Agricultural Research, Avenue Ennasr, BP 415 Rabat Principale, Rabat 10090, Morocco; samira.elotmani@inra.ma (S.E.O.); mouad.chentouf@inra.ma (M.C.); 2 Department of Veterinary Management of Animal Resources, FARAH, Faculty of Veterinary Medicine, University of Liège, 4000 Liège, Belgium; jlhornick@uliege.be (J.-L.H.); jfcabaraux@uliege.be (J.-F.C.); 3 Department of Animal Production and Pastoralism, Ecole Nationale d’Agriculture de Meknès, Meknes 50001, Morocco; akeli@enameknes.ac.ma; 4 Precision Livestock and Nutrition Unit, TERRA Teaching and Research Center, Gembloux Agro-Bio Tech, University of Liège, 5030 Gembloux, Belgium; jerome.bindelle@uliege.be

**Keywords:** GPS collar, sensor, grazing activity, goat, Mediterranean region, forest rangeland

## Abstract

The Global Positioning System (GPS) and sensors technologies are increasingly used to study the grazing behavior of animals. This work was conducted to understand the grazing behavior and energy balance of goats browsing in forest rangeland using GPS and sensors technologies. Forage availability was estimated using the quadrat method during three grazing seasons. Simultaneously, eight indigenous goats were selected to explore their feeding behavior, grazing activities, and energy requirements. The experimental goats were fitted with GPS collars and leg sensors to monitor their grazing activities. At the same time, direct observation was used as a method to study their feeding behavior. Forage availability was higher during spring compared to the summer and autumn seasons. Goats recorded the highest biting rate during summer and autumn (about 22 bites/min). The highest intake rate was recorded during spring (5.6 g DM/min). During spring, goats spent most of their time on grazing (48%) in contrast to the summer and autumn (<31%; *p* < 0.001). They prolonged their lying down time in summer at the expense of standing duration. The time devoted exclusively to grazing (eating) was longer in spring. Walking time in summer and autumn was longer than in spring (*p* < 0.001). During summer and autumn, the energy balance of goats under grazing conditions was in deficit. Using GPS collars and leg sensors appears to be a useful and easily replicable method to explore and understand the seasonal changes in the grazing areas and activities of goats in a mountainous region. The results could help goat herders and managers to develop feeding and grazing systems while increasing the performance of goats in the Mediterranean forest rangeland.

## 1. Introduction

Forest rangelands are an important component of extensive goat production systems in the Mediterranean basin. The pasture production in these areas is characterized by its seasonal variation, mainly on the southern side. For example, in northern Morocco, forage availability records higher values during spring (>2500 kg DM/ha) compared to the summer and autumn (<1700 kg DM/ha) [1]. In terms of goat population, Morocco occupies the third position in the Mediterranean region with 5.2 million heads [2]. The predominance of goats is due to their adaptation to the mountainous topography and the existing forest vegetation. The diet requirements of animals are mainly provided by forest rangelands (about 80%) in the mountainous region of the High Atlas and northern Morocco. Seventy percent of the nation’s goats graze on forest rangeland, i.e., approximately 4 million heads [3]. In northern Morocco, the extensive livestock system is mainly associated with goat herds. Their number is about 627,000 heads concentrated in mountainous areas of the region [4]. Forest rangelands guarantee free year-round feed resources for grazing goats. In addition, extensive goat farming plays a very important socio-economic role for local farmers because it contributes between 68% and 100% to their agricultural incomes [5].

Grazing in forest rangeland is associated with different daily activities compared with those of confined animals, such as time spent browsing and walking. These grazing activities generate additional energy costs due to increased muscular efforts, which could limit the use of the energy available for maintenance and production [6,7]. Moreover, the mountainous topography of the woodland imposes an additional physical activity required for the vertical locomotion of goats. These ascent and descent movements may result in an increase in the time and energy cost necessary to walk a given distance [8]. According to Lachica and Aguilera [9], energy is a key component in animal production. The energy balance (the equilibrium between offered and consumed energy) is essential for developing and maintaining a sustainable animal farming system.

Currently, to our knowledge, despite various studies focused on the feeding behavior of goats in the Mediterranean countries [10,11,12,13], there is limited information in the literature concerning the grazing activities and energy balance of goats in the mountainous forest rangelands. Such information is difficult to obtain only by direct observation because the observer could not accurately measure the individual behavior of animals, such as movement and activity patterns. However, data on animal behavioral activities are essential for understanding their feeding behavior and their interaction with the environment in order to define optimal management intervention strategies [14].

The recent development of the Global Positioning System (GPS) and the increasing availability of sensors technologies to monitor and record behavioral activities provide a real opportunity to extend our database and to understand the grazing behavior of animals [15]. The previous studies on monitoring the grazing activity of animals using sensors and GPS technologies have focused mainly on grazing cattle and sheep [16,17,18,19,20]. To our knowledge, there is no study using these technologies simultaneously to investigate the grazing behavior of goats in the Mediterranean forest rangeland.

The purpose of the research was to ensure the sustainability of the extensive goat production systems through improving the knowledge on grazing behavior and energy expenditure by using GPS collars and sensors, in order to develop targeted decisions to enhance feeding and grazing strategies. To reach our scientific and utilitarian goals, this study was undertaken in mountainous forest rangeland of the southern Mediterranean region (northern Morocco) to examine the effects of the seasonal changes on forage availability, feeding behavior, grazing activities, and energy balance of indigenous goats under grazing conditions.

## 2. Materials and Methods

### 2.1. Study Area

**Figure 1:**
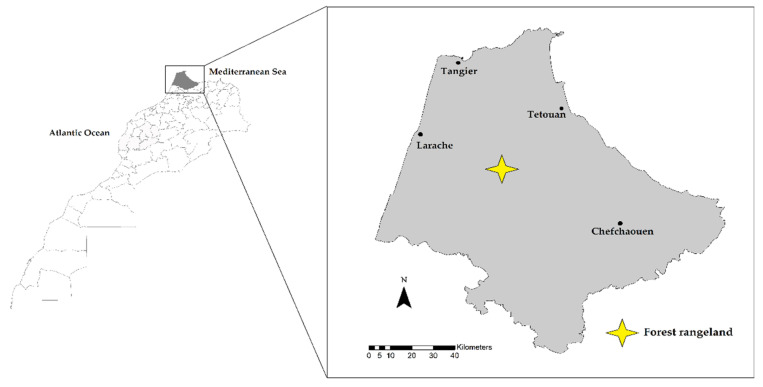
Location of the studied forest rangeland (northern Morocco).

The study was conducted on forested rangeland of Beni Arouss, Rif region, northern Morocco (Figure 1). This woodland is located at an altitude ranging between 250 and 550 m a.s.l (35°18′ N; 5°34′ W). The climate is Mediterranean (rainy and cold in winter and mild in summer), with an annual rainfall varying between 400- and 700-mm. Temperatures range from 3 to 14 °C in winter and from 18 to 38 °C in summer (minimum and maximum, respectively) [21]. The studied pasture is a Mediterranean forest rangeland with vegetation units that go from low formations of *Cistus* species (inclusive of *C. crispus*, *C. monspeliensis*, and *C. salviifolius*) to the high oak groves. Tree vegetation includes *Quercus spp.* (inclusive of *Q. coccifera, Q. ilex*, and *Q. suber*) associated with shrublands dominated by *Arbutus unedo*, *Cistus* spp., and *Erica arborea* [22,23].

### 2.2. Pasture Production

Pasture production was evaluated to know the amount of forage that the grazing animals had at their disposal. Hence, forage availability of selected plants species by goats was estimated using the quadrat method. Therefore, forty quadrats of 40 m^2^ (4 m × 10 m) for woody species and forty quadrats of 1 m^2^ (1 m × 1 m) for herbaceous species (each one embedded within one woody quadrat) were installed seasonally in the studied forest rangeland [1]. The collected biomass samples were oven-dried at 55 °C until having a constant weight to obtain the dry matter (DM).

### 2.3. Data Collection of Feeding Behavior

Eight indigenous meat goats of the Beni Arouss breed (30 ± 2.6 kg live-weight (LW) and 36 ± 6 months of age; in the beginning of the experiment) were chosen for the study from a local goat flock composed of 79 animals browsing in a forest rangeland. Goats spend most of the day on forest pastures. At the end of the grazing day, they are confined in a closed and semi-opened shed inside the farm, which is located on the border of the studied forest rangeland. In northern Morocco, Beni Arouss goat is the only recognized indigenous goat breed. This meat breed uses mountainous forest vegetation exclusively as feed resources. Therefore, the produced meat is highly appreciated by consumers. The daily milk production of the studied group of goats was estimated to 0.5 kg/goat during the lactation period. In addition, this breed shows excellent tolerance to diseases [24].

The experimental goats in the study were chosen based on their similar physiological state. They had been naturally mated at the beginning of August (summer). Thus, the last two months of pregnancy coincided with the months of November and December (autumn), and the kidding in early January (winter). These experimental goats were lactating during the winter and spring.

The forest rangeland was grazed during all seasons, except winter when they are kept in the shed. During winter, forest rangelands’ access is very limited and it corresponds to the kidding period. To guarantee feeding goats during this period, herders trim evergreen tree branches in the forest as forage and bring them to the goat shed [1]. Livestock watering is guaranteed by water sources and streams inside the grazed forest rangeland. The movement and grazing areas of goats browsing in forest rangelands of northern Morocco are increasingly affected by the agricultural expansion. The cultivation plots inside these rangelands are the main barriers restricting goat’s movement and reducing grazing areas [21].

The feeding behavior was assessed by the direct observation method to estimate the bite count and botanical composition of the selected diet. The observation procedure was realized during three consecutive grazing days over three seasons: spring (green season), summer (dry season), and autumn. Data were collected by observing each goat for 10 min, thrice a day (morning, mid-day, and afternoon) making a total of 72 observations per season (3 days × 3 times of the day × 8 animals). Before starting the observation procedure, observers spent several days with the goats to accustom them to the presence of strangers [5,25]. The adaptation (familiarization) procedure was considered as successful when the observer could be as close as possible to the animal (0.5 to 1.5 m) without disturbing their browsing behavior [19]. Trained observers recorded the selected plant species and the number of bites per consumed plant part, which allowed generating the total number of bites (TB). To estimate the average mass per bite, the observer mimicked the bite weight (BW, g DM) for each plant species selected by goats (only the consumed parts of the plant) using the hand-plucking procedure (100 simulated plucks per plant per season) [26]. The hand-plucked samples were collected in special bags and taken to the laboratory in order to determine the average BW of each consumed species. This procedure allowed for estimation of the short-term intake rate (IR, g DM/min) [20]. The IR was calculated as

IR = BR × BW,(1)

where BR is the bite rate (BR, n/min). The daily forage intake (g DM/day) is calculated as the product of total grazing time and intake rate per minute of grazing activity. The grazing activity (eating) involves the acquisition of herbage into the mouth, its mastication, and subsequent swallowing [27].

### 2.4. Grazing Activity Measurements

#### 2.4.1. GPS Collars and IceTag Sensor Data

**Figure 2:**
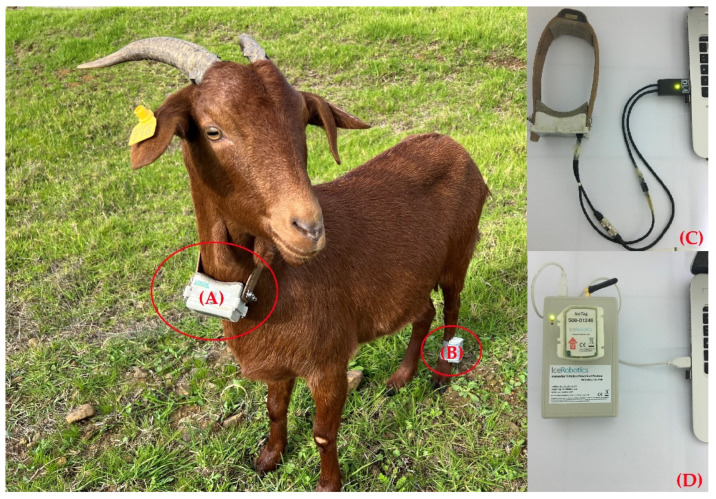
Indigenous goat of Beni Arouss fitted with GPS collar on the neck (**A**) and IceTag sensor on the rear left leg (**B**). Downloading GPS collar (**C**) and IceTag (**D**) data.

The activities of goats, under grazing conditions, were characterized according to the methods described by Goetsch et al. [28]. The grazing activity measurements were simultaneously realized with the feeding behavior study on the same eight experimental goats. Each goat was fitted with GPS collar (Model 3300SL; Lotek Wireless, Newmarket, ON, Canada) on the neck and IceTag sensor on the rear left leg (IceRobotics Ltd., Scotland, UK) for three days. Several days before the real measurements, these goats were equipped with GPS collars and IceTag sensors to accustom them to the attached devices on their body (Figure 2). GPS data were used to estimate the location, speed, horizontal, and vertical traveled distances. Data were analyzed by GPS3000 Host software (Lotek Wireless, Newmarket, ON, Canada). Coordinates were converted from UTM WGS84 to Moroccan Transverse Mercator using ArcGIS 10.× (ESRI, Redlands, CA, USA). The coordinates (x and y) in meters were calculated for each fix record using ArcMap. The horizontal distance (D_H_) between consecutive fixes (x_1_/y_1_; x_2_/y_2_) was calculated as

(2) $$D_{H}=\sqrt{{\left(x_{2}-x_{1}\right)}^{2}+{\left(y_{2}-y_{1}\right)}^{2}}$$

The vertical distance (D_V_) was derived from the difference in altitude between successive positions 1 (z_1_) and 2 (z_2_). The speed of grazing goats was calculated dividing the total distance “D_T_” by the time elapsed between two successive positions, where

(3) $$D_{T}=\sqrt{{\left(D_{H}\right)}^{2}+{\left(D_{V}\right)}^{2}}$$

The IceTag data were analyzed by IceManager software (IceRobotics Ltd., Scotland, UK). The variables provided are lying (sitting to rest or ruminate), standing (standing without eating and when ruminating), number of steps and motion index (a proprietary metric of the overall leg activity as measured in three dimensions). The total standing time includes both grazing and non-grazing periods. Lying time is solely or predominantly without grazing. The collars were scheduled to acquire a GPS fix every 5 min, then the data from IceTags were also converted to the same intervals. A calibration study and classification tree analysis were used to predict grazing activities of goats (grazing/eating, resting while lying, resting while standing and walking without grazing (head up and moving from one place to another)) in 5 min intervals as described by Beker et al. [29] and Brassard et al. [30]. The classification and regression tree (CART) analysis was used to construct the classification trees using motion index, number of steps, time spent standing and lying from IceTag sensors and x-activity, y-activity, head-down, and traveled horizontal distance from the GPS collars as predictor variables; grazing activity was considered as the target variable.

The grazing area was calculated in ArcGIS based on polygon resulting from buffers of 15 m width to both sides of each fitted goat with GPS collar [31].

#### 2.4.2. Weather Data

During days of behavior data collection, the ambient environment temperature (Ta) and relative humidity (RH) were monitored every 5 min along the itinerary of grazing goats with a digital thermo-hygrometer (608-H2; Testo, Germany). The temperature–humidity index (THI) was calculated as [32]

THI = 0.8 × Ta + (RH × (Ta − 14.4) ÷ 100) + 46.6(4)

THI values of 74 or less are considered as comfortable (absence of heat stress). However, values between 75 and 78 are considered as stressful, and between 79 and 83 as severe heat stress. When values are higher than 84, animals experience extreme heat stress causing high distress, and animals are unable to maintain thermoregulatory mechanisms or normal temperature [33].

### 2.5. Energy Requirements and Balance of Grazing Goats

The daily metabolizable energy requirement for maintenance (MEm) of goats was estimated to 424.2 kJ/kg LW^0.75^ [34]. In order to calculate the energy expenditure for locomotion, the equation proposed by Lachica and Aguilera [9] was used with 3.35 and 31.7 J/kg LW per meter for horizontal and vertical (ascend) displacement, respectively. No energy recovery for vertical descent was applied, assuming that such expenditure was similar to that for horizontal walk, as stated by NRC [34]. During pregnancy period, NRC [34] recommended an additional 318 kJ/kg LW^0.75^ of the metabolizable energy (ME) in the last 2 months of gestation and a 20% greater value was suggested for does with more than one goat kid. On that point, pregnancy ME requirements were added during the autumn season, corresponding to the period of late pregnancy. The additional requirement energy of milk production was estimated according to NRC [34]. Knowing that the fat milk composition of Beni Arouss goats averages 3% [35], the additional requirement energy for lactation was estimated to 5020 kJ/kg of milk, including requirements for nursing kids. This energy requirement for milk production was added during spring, corresponding to the lactation period of the experimental goats.

The ME of each ingested plant species by goats was taken from Chebli et al. [36], who studied the seasonal variations in chemical composition, in vitro digestibility, and ME of plant species browsed by goats, during the same year and seasons, in the same forest rangeland of northern Morocco.

### 2.6. Statistical Analysis

Data analyses were performed using SAS software (SAS version 9.4, SAS Inst. Cary, NC, USA) [37]. Before analyses, data expressed as percentage were arcsine-square root-transformed to normalize their distribution [38]. Feeding behavior (BR and IR) and grazing activity data were analyzed according to the PROC MIXED procedure of SAS [39] with the daily observation on each goat as experimental unit. Feeding behavior and grazing activity parameters were compared across seasons (i.e., spring, summer, and autumn). The individual goat was considered as a random factor to prevent this variance from being incorporated in the error term of the analysis. For all analyses, the significance level was declared at *p* < 0.05. In case of significant effect, means were compared using the Tukey’s test.

## 3. Results

### 3.1. Forage Availability and Feeding Behavior

Three distinct groups of plant species dominated the forest vegetation and composed the bulk of the goats’ diet, namely: shrubs (*Arbutus unedo*, *Calicotome villosa*, *Cistus* spp., *Erica arborea*, *Lavandula stoechas*, *Myrtus communis*, *Phillyrea media*, *Pistacia lentiscus*, and *Rubus ulmifolius*), herbaceous (*Anthemis cotula*, *Brachypodium distachyon*, *Bromus rigidus*, *Calamintha nepeta*, *Cynodon dactylon*, *Eryngium tricuspidatum*, *Lythrum junceum*, and *Rumex bucephalophorus*), and trees (*Quercus* spp. and *Olea europaea* subsp. *O. europaea* var. *sylvestris*). The outcomes for the seasonal variation in forage production of the studied forest rangeland and the feeding behavior of the experimental grazing goats are presented in Table 1. There were significant differences in forage availability within each measurement season (*p* < 0.001). The forage production decreased significantly by 31% during summer and 47% during autumn compared to the spring season. For feeding behavior parameters, the season affected the average bite and intake rates (*p* < 0.05). On the one hand, the highest biting rate was recorded equally during summer and autumn (about 22 bites/min). On the other hand, the highest intake rate was recorded during spring (5.57 g DM/min).

### 3.2. Temperature–Humidity Index

**Figure 3:**
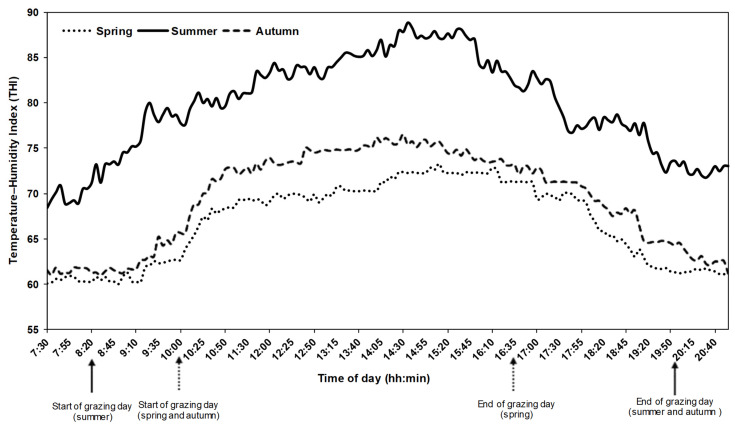
Seasonal changes in the hourly temperature–humidity index during the observation days of goats grazing in a Mediterranean forest rangeland of northern Morocco (local time: GMT + 1).

The mean of daily RH values ranged from 11 to 40, 33 to 79, and 17 to 60% in spring, summer, and autumn, respectively. The mean of daily Ta values ranged between 17 and 31, 22 and 37, and 20 and 32 °C in spring, summer, and autumn, respectively. Hence, the mean daily THI was 66-, 69-, and 80-units during spring, autumn, and summer, respectively. The highest values of THI in different seasons were recorded between 12 h and 16 h (middle of the day) as displayed in Figure 3.

### 3.3. Grazing Activities

Table 2 summarizes the seasonal variation in the grazing activities of goats. Goats spent the majority of their daytime foraging budget grazing (including actual forage prehension, searching, and walking) during spring and autumn (*p* < 0.001). The goats prolonged their lying time in summer (*p* < 0.001) at the expense of standing duration. The number of steps was numerically similar and significantly greater in both seasons of summer and autumn (*p* < 0.001). The analysis of GPS collar data showed a significant effect of season on the measured parameters (*p* < 0.001). The horizontal distance traveled by goats was similar and significantly higher in autumn and summer. A similar tendency was observed for vertical distance. Conversely, the speed of goats was significantly higher in spring compared to the other seasons (*p* < 0.001). The duration of the foraging day (time spent on the pasture) was prolonged (*p* < 0.001) in summer when compared to autumn, and spring (*p* < 0.001). According to the CART analysis, the time spent actually grazing (eating) was longer in spring and similar in summer and autumn (*p* < 0.001). Resting while standing was similar between seasons (*p* = 0.191). The time spent walking without grazing (eating) ranked as follows: autumn > summer > spring (*p* < 0.001). Based on the result of GPS collars and ArcGIS, the grazing area was estimated to be 8.3, 17.6, and 16.3 ha in spring, summer, and autumn, respectively.

### 3.4. Energy Requirements and Balance of Grazing Goats

**Figure 4:**
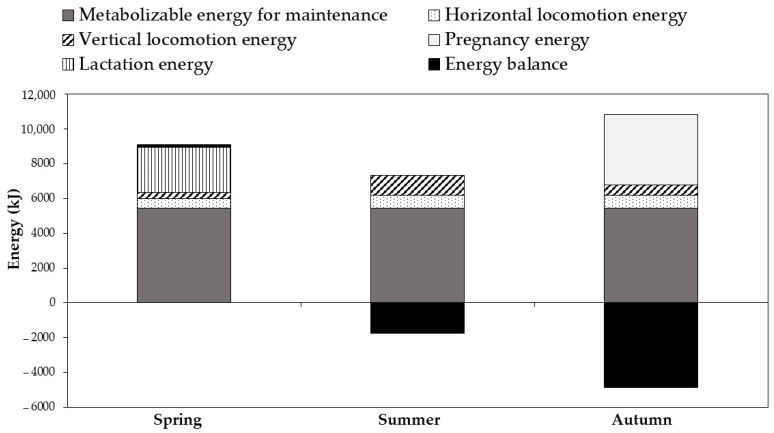
Estimation of the seasonal energy requirements (metabolizable energy for maintenance, locomotion, lactation, and pregnancy) and the energy balance of the experimental goats grazing in a Mediterranean forest rangeland of northern Morocco.

Figure 4 shows the estimation of the seasonal energy requirements and the energy balance of grazing goats. Energy requirements were significantly affected by the season (*p* < 0.01). The highest ME intake was recorded during spring (9086 kJ/day), in contrast to summer (5566 kJ/day) and autumn (5978 kJ/day). The energy expenditure for horizontal locomotion was higher and similar during summer and autumn (about 750 kJ/day) than in spring (565 kJ/day). The low energy expenditure for vertical locomotion was observed in the green season (359 kJ/day). The pregnancy ME requirements were estimated to be 4076 kJ/day in autumn. The additional requirement energy for lactation during spring was estimated at 2531 kJ/day. Consequently, a low surplus of energy, under these grazing conditions, was recorded during the green season (<2%), conversely to the summer and autumn seasons, which recorded a high energy balance deficit of 24 and 45%, respectively.

## 4. Discussion

### 4.1. Forage Availability and Feeding Behavior

The pasture production measurements revealed that shrubs (mainly *A. unedo, Cistus* spp., and *E. arborea*) are the most available forage species for goats in the study area. Shrubs’ contribution to the forage availability was about 90% during the three studied seasons. For herbaceous and trees, their contributions varied across season from 2 to 9%, and from 0.5 to 2%, respectively. Similar findings were reported by several authors who studied the botanical composition and forage availability of forest rangeland in northern Morocco [5,40]. This forage availability depended considerably on the season and the botanical composition of the rangeland. The high forage availability recorded in the spring could be explained by the vegetation cycle, which corresponds to the green season and to more favorable weather conditions for the growth of plants in comparison to summer and autumn. In the Mediterranean forest rangeland of Greece, the strong seasonality of forage availability was also confirmed, even for woody species that are usually less impacted by the change in season [13].

The seasonality of feeding behavior was confirmed by several studies conducted in the Monte Desert of Argentina [41], South African woodland [42], and Greek woodland [13]. In agreement with Barros et al. [43], goats recorded faster bite rate in the summer and autumn in order to maximize their intake rate due to the decrease in forage availability. Papachristou [44] also signaled the increase in bite rate from spring to summer similarly to the current finding.

### 4.2. Grazing Activities

The grazing activities of goats vary throughout the three studied seasons. Several authors [45,46,47] confirmed this finding. Safari et al. [46] reported that goats in the semi-arid area of Tanzania increased their grazing (eating) time (57–68%) and decreased standing time (6.8–1.4%) from the rainy to late summer, while their time spent walking was similar (27%). In a similar region of Zimbabwe, goats spent most of the time eating in the wet season (52–75%) in contrast to the summer (29–50%) [45]. Similarly to the current results, goats spent 48% of their feeding duration on grazing (eating) during the green season in contrast to the summer (27%) and autumn (31%) seasons. This result could be explained by the high abundance of preferred shrubs (*Cistus* spp. and *L. stoechas*) and herbaceous plants during the green season. Similarly, Zampaligré and Schlecht [48] reported that the abundance of preferred species by goats increased the reserved time for grazing. Likewise, the seasonal changes in goats’ grazing activities could also be explained by the duration of the grazing day recorded during each season, which was shorter in spring and longer during the summer and autumn seasons in the current study. Thus, it could be suspected that to increase the feeding intake of the goats, the duration of the grazing day was extended to recover lost time that was allocated for walking during the autumn, and for lying and walking in the summer. Similarly, Safari et al. [46] and Rodrigues et al. [47] reported that a longer duration of the grazing day was observed in goats grazing during seasons of low forage availability. In summer, the time dedicated to lying was observed mainly during the middle of the day because goats prefer lying under shady trees when the sun is highest. The current results agreed with the research of Ferreira et al. [49] and Tolu et al. [50] who reported that the small ruminants are more active in the morning and during the afternoon, whereas in the middle of the day they prefer to rest lying down. In the semi-arid area of Tanzania, Safari et al. [46] declared that goats extended the duration of their grazing day in the summer compared to the rainy season to meet their intake requirements.

Additionally, declined grazing (eating) time could also be linked to the increasingly stressful conditions (THI > 75) recorded during the summer and autumn, mainly in the middle of the day. As declared by Askar et al. [51], air temperature and THI affected the temporal pattern in the grazing time of goats. Tovar-Luna et al. [52] and Askar et al. [51] also reported relatively high percentages of time spent grazing when the temperature and THI had started to decline. As announced by McDowell [53] and Goetsch [54], in a high THI environment, goats reduce their intake rate and increase their watering to regulate their body temperature. Consequently, the dry matter intake decreases significantly following exposure to heat stress [55]. Such results suggest that goats should graze during the periods of the milder weather of the day to better benefit from pastures and implicitly increase their dry matter intake.

The extended walking duration could also be responsible for the reduced grazing time. According to Baumont et al. [56], walking is associated with forage search and selection. The green season is characterized by high forage diversity compared to other seasons; therefore, goats did not have to walk a long distance to graze plant species, resulting in a reduced walking percentage. The autumn and summer seasons are characterized by low forage availability, which meant goats were walking for longer durations in search of palatable species. Similarly, Schlecht et al. [31] reported the seasonal variation in the journey length of goats due to the change in forage availability in the mountain range of northern Oman. It could be assumed that the large walking duration estimated during the summer and autumn could be associated with the forage availability. Furthermore, goats prefer to move further towards higher elevations where the temperature is lower, and forage is more available (not previously grazed zone). In accordance with many relevant studies, forage availability is the major determinant of grazing activities [31,48]. The higher daily proportion of time spent walking could explain the high number of steps and the long daily distance covered by goats during autumn and summer.

### 4.3. Seasonal Fulfillment of Feed and Energy Requirements of Goats under Grazing Conditions

Due to the considerable traveled distances and altitudinal displacement, goats spent a significant energy expenditure at mountainous pasture in contrast to goats in the flat rangeland. Based on the feeding behavior and grazing activities data, the daily DM intake of goats was estimated at 84, 68, and 70 g/kg LW^0.75^ in spring, summer, and autumn, respectively. The results of the daily DM intake were within the ranges indicated elsewhere by Luo et al. [57] and Dove [58] for meat goats. The average ME of maintenance (without locomotion, lactation, and pregnancy energy requirements) needed for a goat in the present case study was estimated at 5438 kJ/goat/day. Considering the physical (locomotion) and physiological states (pregnancy and lactation) of grazing goats, it could be assumed that goats met their average energy needs at 101% in spring, 76% in summer, and 55% (or 88% if a goat is not pregnant) in autumn. Consequently, goats meet their requirements in terms of energy only during the green season, in contrast to grazing in summer and autumn, where the energy balance was negative. This energy deficit translated into the net weight loss of goats during summer and autumn, and also the high abortion rate, observed during the experimentation and in the experiment flock and other neighboring farms where the forest rangeland is the only feed source of grazing goats. Moreover, the non-satisfying of the feed requirements is revealed in low milk production, which implicitly does not meet the nutritional needs of the goats’ kids who need on average between 0.5 and 1.5 L during the first three months of age, which causes neonatal mortality or having weak kids with low growth performance. The studied forest rangeland is browsed by one flock of 79 goats, giving thus an estimated stocking rate of 4.2, 2.9, and 3 goats/ha in spring, summer, and autumn, respectively. The forage availability varies over time depending on the climate of the year, the phenological stage of the associated species, and losses not due to the grazing (leaf fall, attacks from insects, trampling, etc.). Only a certain proportion could be consumed by animals. For this study, we assumed that the DM loss is about 50%. According to the results of forage availability and ME of plant species data [36], the studied forest rangeland supplied 13,271, 7166, and 5359 MJ ME/ha in spring, summer, and autumn, respectively. A stocking rate of 4.1, 2.6, and 1.4 goats/ha should be applied to guarantee the energy equilibrium of goats during spring, summer, and autumn, respectively. Consequently, in order to meet the energy requirements of goats, it would be necessary to reduce their number per hectare to ensure the goat farm’s and forest resource’s sustainability. Moreover, an alternative solution could be considered through goats’ food supplementation during forage gaps and drought periods if the herder plans to maintain high animal numbers in the forest rangeland.

In Doñana natural park of south-western Spain, it was stated that grazing met the average energy requirements of goats to 112%, 102%, and 65% during spring, summer, and autumn, respectively [59]. The estimated energy balance surplus in Doñana natural park could be due to their topography (no vertical locomotion: null slope), pasture quality, and the duration of grazing time. The specific differences observed during spring and autumn could be attributed to the physiological states of goats (pregnancy and lactation), which were not considered in this study. Furthermore, as reported by Moe et al. [60], it should be noted that the estimated values of the energy requirement of animals could be improved by considering several factors, which increase energy expended for maintenance such as environmental stress, physiological state, nutrient imbalances, disease, and tissue energy gain.

## 5. Conclusions

Data on individual behavior, such as movement and activity patterns, are often important for the management of grazing animals. The forage availability of the studied forest rangeland is currently known. In addition, it was shown that the higher biting rate leads to increase short-term intake rates. The combination of GPS collars and leg sensors proved very useful to explore the grazing areas and activities of meat goats in southern Mediterranean forest rangelands. An estimated deficit of the daily energy requirements was noted during the summer and autumn. The results emphasize a high stocking rate during summer and autumn. To meet the energy requirements of goats, it is necessary to develop feeding strategies and grazing management in order to enhance, simultaneously, the production performance of grazing goats and the durability of forest resources. The results could provide useful and target information for herders to elaborate supplementary diet formulation considering grazing activities and the quality of consumed species. The scope of the research carried out could also be implemented in the case of goats reared mainly for milk. Overall, these findings could be used as the first guide for future studies and managers interested in the feeding and grazing behavior of goats.

## Figures and Tables

**Table 1:** Seasonal variation in forage availability (kg DM/ha), bite rate (bites/min), and intake rate (g DM/min) of goats grazing in a Mediterranean forest rangeland of northern Morocco.

Item	Forage Availability	Bite Rate	Intake Rate
Spring	3143 ^a^	17.7 ^b^	5.57 ^a^
Summer	2175 ^b^	21.9 ^a^	4.52 ^b^
Autumn	1662 ^c^	22.1 ^a^	4.94 ^b^
SEM	53.2	0.519	0.330
*p*-Value	<0.001	0.015	0.013

SEM, standard error of the mean. Means with different superscripts (a–c) within the column indicate significant differences (*p* < 0.05).

**Table 2:** Seasonal variation in grazing activities of goats in a Mediterranean forest rangeland of northern Morocco.

Item	Spring	Summer	Autumn	SEM	*p*-Value
**IceTag data**					
Lying down (%)	3.10 ^b^	15.4 ^a^	2.10 ^b^	0.447	<0.001
Standing ^1^ (%)	96.9 ^a^	84.6 ^b^	97.9 ^a^	0.850	<0.001
Steps (×1000)	7.61 ^b^	11.3 ^a^	11.4 ^a^	0.279	<0.001
**GPS collar data**					
Horizontal distance (km/day)	5.62 ^b^	7.43 ^a^	7.55 ^a^	0.114	<0.001
Vertical distance (km/day)	0.377 ^c^	1.21 ^a^	0.595 ^b^	0.044	<0.001
Speed (m/s)	0.238 ^a^	0.156 ^c^	0.215 ^b^	0.004	<0.001
Duration of grazing day (min/day)	398 ^c^	708 ^a^	593 ^b^	19.9	<0.001
**CART analysis data (%)**					
Grazing (eating)	48.4 ^a^	27.1 ^b^	30.5 ^b^	0.990	<0.001
Resting while standing	25.4	21.4	20.5	0.896	0.191
Walking without grazing	23.1 ^c^	36.1 ^b^	46.9 ^a^	0.799	<0.001

SEM, standard error of the mean. Means with different superscripts within the row indicate significant differences (*p* < 0.05). ^1^ Standing includes grazing, resting while standing, and walking without grazing times.

## Data Availability

The data presented in this study are available from the corresponding author on request.
